# A qualitative dual-site analysis of the pharmacist discharge care (PHARM-DC) intervention using the CFIR framework

**DOI:** 10.1186/s12913-022-07583-5

**Published:** 2022-02-12

**Authors:** Logan T. Murry, Michelle S. Keller, Joshua M. Pevnick, Jeffrey L. Schnipper, Korey A. Kennelty, for the PHARM-DC Group, An T. Nguyen, Andrew Henreid, Jesse Wisniewski, Kallie Amer, Christine Armbruster, Nicole Conti, James Guan, Shirley Wu, Donna W. Leang, Ruby Llamas-Sandoval, Emily Phung, Olga Rosen, Sonja L. Rosen, Audrienne Salandanan, Rita Shane, Eun Ji Michelle Ko, Dylan Moriarty, Anne Marie Muske, Lina Matta, John Fanikos

**Affiliations:** 1 Department of Pharmacy Practice, The University of Iowa College of Pharmacy, 180 S Grand, Iowa City, IA 52242 USA; 2 Division of General Internal Medicine, Department of Medicine, Cedars-Sinai Medical Center, Los Angeles, CA USA; 3 Division of Informatics, Department of Biomedical Sciences, Cedars-Sinai Medical Center, Los Angeles, CA USA; 4 Department of Health Policy and Management, UCLA Fielding School of Public Health, Los Angeles, CA USA; 5 Brigham Health Hospital Medicine Unit, Division of General Internal Medicine and Primary Care, Brigham and Women’s Hospital, Boston, MA USA; 6 Harvard Medical School, Boston, MA USA

**Keywords:** Transitions-of-care, Post-discharge adverse drug events, Medication related hospital readmission, Pharmacy

## Abstract

**Introduction:**

Older adults face several challenges when transitioning from acute hospitals to community-based care. The PHARMacist Discharge Care (PHARM-DC) intervention is a pharmacist-led Transitions of Care (TOC) program intended to reduce 30-day hospital readmissions and emergency department visits at two large hospitals. This study used the Consolidated Framework for Implementation Research (CFIR) framework to evaluate pharmacist perceptions of the PHARM-DC intervention.

**Methods:**

Intervention pharmacists and pharmacy administrators were purposively recruited by study team members located within each participating institution. Study team members located within each institution coordinated with two study authors unaffiliated with the institutions implementing the intervention to conduct interviews and focus groups remotely via telecommunication software. Interviews were recorded and transcribed, with transcriptions imported into NVivo for qualitative analysis. Qualitative analysis was performed using an iterative process to identify “a priori” constructs based on CFIR domains (intervention characteristics, outer setting, inner setting, characteristics of the individuals involved, and the process of implementation) and to create overarching themes as identified during coding.

**Results:**

In total, ten semi-structured interviews and one focus group were completed across both hospitals. At Site A, six interviews were conducted with intervention pharmacists and pharmacists in administrative roles. Also at Site A, one focus group comprised of five intervention pharmacists was conducted. At Site B, interviews were conducted with four intervention pharmacists and pharmacists in administrative roles. Three overarching themes were identified: PHARM-DC and Institutional Context, Importance of PHARM-DC Adaptability, and Recommendations for PHARM-DC Improvement and Sustainability. Increasing pharmacist support for technical tasks and navigating pharmacist-patient language barriers were important to intervention implementation and delivery. Identifying cost-savings and quantifying outcomes as a result of the intervention were particularly important when considering how to sustain and expand the PHARM-DC intervention.

**Conclusion:**

The PHARM-DC intervention can successfully be implemented at two institutions with considerable variations in TOC initiatives, resources, and staffing. Future implementation of PHARM-DC interventions should consider the themes identified, including an examination of institution-specific contextual factors such as the roles that pharmacy technicians may play in TOC interventions, the importance of intervention adaptability to account for patient needs and institutional resources, and pharmacist recommendations for intervention improvement and sustainability.

**Trial registration:**

NCT04071951.

**Supplementary Information:**

The online version contains supplementary material available at 10.1186/s12913-022-07583-5.

## Background

Adverse drug events (ADEs) have negative effects on patient health outcomes after discharge and during transitions of care (TOC), with 17–19% of older adults experiencing an ADE post-discharge and ADEs contributing to 23–38% of hospital readmissions in older adults [1–4]. A number of risk factors have been implicated in medication-related admissions and readmissions [5], including but not limited to: absent or inadequate medication reconciliation [6–8], lack of pharmacy consultation during the inpatient stay [9], polypharmacy [10, 11], medication non-adherence [12], and discharge to non-home settings [13, 14].

Recent trials on the effectiveness of pharmacy-led TOC initiatives have used a variety of pharmacy-led intervention components with mixed results. The Medication Reviews Bridging Healthcare (MedBridge) trial conducted by Kempen et al. [15] evaluated the effect of two distinct pharmacist-led interventions, a comprehensive medication review (CMR) and CMR with detailed and structured post discharge follow-up, on the primary endpoint of emergency department attendances and admissions. The results of the MedBridge trial failed to show a significant difference between standard care and intervention groups. Similarly, the Optimizing Therapy to Prevent Avoidable Hospital Admissions in the Multimorbid Older People (OPERAM) cluster randomized control trial conducted by Blum et al [16]. evaluated the effect of a structured pharmacotherapy optimization intervention performed jointly by a physician and a pharmacist, with the support of clinic decision software used to screen prescription medications and evaluate potentially inappropriate medications. The study also failed to show a statistical difference between control and intervention groups on the primary outcomes of drug related hospital admission within 12 months. Conversely, the Odense Pharmacist Trial Investigating Medication Interventions at Sector Transfer (OPTIMIST) trial conducted by Ravn-Nielsen et al. [17] found a significant positive effect on readmission or emergency department attendance within 180 days for individuals who received an extended version of the intervention. The trial consisted of three trial arms: usual pharmaceutical care (control), basic intervention, or extended intervention. Both the basic and extended intervention included a pharmacist-delivered medication review and motivational interviewing (MI), with the extended intervention including additional elements of medication reconciliation at discharge, a structured motivational interview with the patient, a report of medication changes for the patient’s primary care physician, and a telephone follow-up within 3 days of discharge. Overall, the evidence for pharmacy-led TOC interventions is currently mixed, with successful interventions including MI and medication review. Given the limited and mixed evidence on pharmacy TOC interventions and the considerable variations in the offerings of pharmacy-led TOC interventions, there is additional need to develop and evaluate additional pharmacy-led TOC interventions.

One ongoing trial of a pharmacy-led TOC intervention is the PHARMacist Discharge Care intervention (PHARM-DC), which provides patients with TOC services including but not limited to: medication reconciliation upon admission, discharge medication reconciliation, medication regimen review, patient education and counseling during and after discharge, and bedside medication delivery. A more thorough description of the PHARM-DC intervention is included subsequently.

The PHARM-DC intervention is currently under evaluation in a pragmatic randomized controlled clinical trial (PRCT) with 1:1 randomization at the patient level at two large hospitals in the Western and Northeastern United States [18]. To understand facilitators and barriers to implementation and sustainability across and between sites, and to enable future successful dissemination of the intervention to other institutions and settings, we undertook a qualitative evaluation using methods informed by implementation science.

## Objectives

The objectives of this study were to: 1) explore intervention pharmacist perspectives of the PHARM-DC intervention and 2) to relate pharmacist perspectives to the Consolidated Framework for Implementation Research (CFIR) framework to understand intervention implementation and sustainability across and between implementation sites.

## Methods

This was a multicenter exploratory qualitative study using interviews and focus groups at two large academic medical centers in the Northeastern and Western United States.

### PHARM-DC intervention

The PHARM-DC intervention is a TOC intervention designed to address factors associated with post-discharge ADEs and medication related hospital readmissions. While both control and intervention patients receive a best possible medication history (BPHM) [19] and medication reconciliation upon admission, the PHARM-DC intervention also provides discharge medication reconciliation; medication regimen review; increased communication with caregivers, inpatient and post-discharge providers, and retail pharmacies; patient education and counseling during and after discharge; bedside medication delivery if indicated; and MI. MI is a guided patient-centered form of counseling that directs patients to reason their way to conclusions that facilitate behavioral change and health goal attainment [20]. The interventions components focused on four specific categories where inpatient pharmacists have the ability to mitigate post-discharge ADEs: medication discrepancies, medication adherence, side effect management, and medication regimen optimization. A full list of intervention components and categorization is included in Table 1 and the intervention is described in more detail elsewhere [18].

**Table 1:** PHARM-DC intervention components and categorization [18]

Action	Timing	Medication Reconciliation	Regimen Review/ Polypharmacy	Side Effect Mgmt.	Adherence
**Pre-Discharge Intervention**	Hospital day 1, or as soon as possible when the patient/caregiver are available to discuss medication issues	Confirm accuracy of admission medication reconciliation	1. Assess regimen appropriateness 2. Talk to inpatient team re: recommended changes 3. Consider trial off meds as inpatient	Discuss past side effects, ways to avoid or treat them	Address most critical barriers to adherence and intervene where possible
**At-Discharge Intervention**	Day of discharge	1. Discharge med rec, communicate with inpatient team to correct any discrepancies 2. Briefly review med changes with patient	Document and communicate recommended medication changes to PCP	Briefly discuss potential side effects, red flags for new meds, what to do if red flags occur	
**Post-Discharge Intervention**	Phone call 1–3 days after discharge	1. Call pharmacy to ensure meds picked up, communicate discontinued medications 2. Call with patient: confirm patient’s regimen. 3.Communicate with PCP re: changes in regimen.		Call with patient: ask about any new side effects, red flags to watch for	1. Call with patient: picked up meds, taking meds. 2. Motivational interviewing. 3. Address other barriers to adherence. 4.Communicate with PCP: tasks done and to be done to improve adherence.

Patients were eligible to receive the PHARM-DC intervention if they met the initial inclusions criteria of 1) admitted to a medical, cardiology, or oncology inpatient service, 2) greater than or equal to 65 years of age, AND 3) on 10 or more chronic medications or three or more high risk-medications (e.g., anticoagulants, antiplatelets, and medications aimed at controlling blood sugar). During this study, the age inclusion criteria was adjusted to allow individuals greater than or equal to 55 years of age to be eligible for the study in order to increase the enrollment rate. Patients in the control group received usual care, which included a BPMH and admission medication reconciliation, standard tools to assist with medication reconciliation built into the electronic medical record, discharge medication counseling by each patient’s nurse, and post-discharge nurse of pharmacist contact if there was operational capacity [21, 22]. Control patients were not prevented from receiving additional pharmacy-led TOC services and support. Currently, the PHARM-DC intervention is undergoing formal evaluation in a dual cite PRCT, testing the effect of the intervention on rate of 30-day post-discharge readmission, observation stay, or emergency department visit [18].

### Settings

Site A is a large health system located in the Western United States with a main medical center capacity of 886 beds. Site A has a Level 1 trauma center and is a major teaching hospital, with Medicare and private insurance comprising the majority of the payer mix in 2015. Site B is a single hospital within a large integrated health care system in the Northeastern United States. Similar to Site A, Site B is a major teaching hospital with 793 beds, with Medicare and private insurance comprising the majority of the payor mix in 2019. Annually, Site A and Site B have approximately 55,000 and 46,000 admissions, respectively. Consistent with NIH requirements, a single IRB associated with Site A was used for this qualitative study as well as the PRCT, with Site B ceding approval authority to the single Institutional Review Board (IRB). The PRCT was granted a waiver of informed consent due to the pragmatic nature of the intervention and the minimal risk to study participants.

The two hospitals involved in the implementation of the PHARM-DC provided some components of the PHARM-DC intervention prior to intervention initiation. For example, both study sites consistently collected BPMHs for selected medical patients admitted to the hospital from the emergency department. Site A collected BPMHs for the majority of patients prior to the PHARM-DC intervention, while Site B collected this information for approximately half of the emergency department admitted patient population. Prior to the PHARM-DC intervention, both sites provided inpatient pharmacist visits, delivery of medications to inpatients prior to discharge (i.e., “Meds to Beds”), and post-discharge pharmacist phone calls to some patients based on clinical or practitioner-identified necessity and based upon pharmacist availability.

### Participants

Participants were purposively recruited by study team members located within each of the study institutions. Participants were identified and selected for participation based on meeting either of two criteria: 1) pharmacy staff providing the PHARM-DC intervention or 2) directly or indirectly supervising pharmacy staff providing the PHARM-DC intervention. Interviews and focus groups were scheduled and conducted by two pharmacy-trained investigators unaffiliated with either study site, with interview and focus group duration scheduled for 30 to 60 min, depending on participant availability and role within the PHARM-DC intervention. Interviews were considered the preferred method of qualitative data collection, but when a site had a larger number of intervention pharmacist with staffing overlap and limited flexibility, focus groups were offered as a potential alternative to facilitate qualitative data collection. Additionally, focus groups comprised of homogenous groups of intervention pharmacists have the potential to improve the overall quality and depth of the information collected, with conversations between and across intervention pharmacists providing insights that may not be captured using interviews alone. Interviews and focus groups were conducted from September 2020 to March 2021. Interviews were conducted using video conferencing software, focus groups were conducted telephonically. All interviews and focus groups were conducted while the larger PRCT evaluating the primary and secondary endpoints of the PHARM-DC intervention was ongoing. Participants did not receive compensation to participate in interviews and focus groups.

### Interview guides

Qualitative data collection guides were developed to facilitate discussion during the interviews and focus groups. The initial interview guides were developed using constructs from CFIR domains to gather data reflecting barriers and facilitators for PHARM-DC intervention implementation and sustainability. For this study, the CFIR domains aligned with the following elements specific to the PHARM-DC intervention: Intervention characteristics (of PHARM-DC), Outer setting (state- and patient-specific factors unique to each intervention site), Inner setting (characteristics and context of each intervention site), Characteristics of individuals (pharmacists involved in the PHARM-DC intervention), and Process (delivering and evaluating the PHARM-DC intervention). Questions were designed and mapped to CFIR domains. Interview guides were iteratively adapted as interviews and focus groups were completed to accommodate new information which further reflected CFIR domains or interview topics that reflected intervention implementation, sustainability, or feasibility. Final qualitative data collection guides for pharmacists providing the intervention and for pharmacy management/administration are included in Additional files 1 and 2, respectively.

### Analysis

An initial deductive Framework Analysis approach [23] was used to map pharmacist responses to CFIR domains. In the initial analysis, two study authors (L.M. and K.A.K.) independently read interview and focus group transcriptions and deductively assigned quotes to CFIR domains. Initial mapping was compared using an iterative approach, with the involved study authors discussing CFIR domains and quotation mapping. After initial mapping was performed, the study authors who performed the initial deductive coding reviewed mapped quotations to identify inductive themes and subthemes that reflected pharmacist perceptions of facilitators and barriers to the PHARM-DC intervention, comparing and contrasting the responses from participants at each site. Once CFIR domains had been assigned to quotations, an inductive coding process was used to determine overarching themes that reflected pharmacist perceptions of PHARM-DC implementation. After inductive themes and subthemes were assigned by two study authors, a third study author involved in the interview and focus group guide generation and familiar with the CFIR domains reviewed the themes and subthemes for biases and accuracy, confirming that coding accurately reflected the experiences of PHARM-DC pharmacists and appropriately and fairly highlighted the facilitators and barriers across and between sites.

## Results

In total, 10 interviews and one focus group were completed across both institutions. At Site A, six interviews were conducted with intervention pharmacists and pharmacists in administrative roles. Also at site A, one focus group comprised of five TOC pharmacists who were currently delivering the PHARM-DC intervention to recruited patients was conducted. The focus group was deemed most appropriate at Site A due to schedule limitations and overlap of staffing amongst the PHARM-DC intervention pharmacists. At Site B, four interviews were conducted with intervention pharmacists and pharmacists in administrative roles. Pharmacists performing the intervention participated in qualitative interviews and focus groups, with a smaller number of pharmacists at Site B providing the intervention.

From qualitative analysis, deductive coding identified representative pharmacist quotations for all of the CFIR domains. Further, two study authors agreed on three overarching themes after the inductive coding process: 1) PHARM-DC and Institutional Contexts, 2) Importance of PHARM-DC adaptability, and 3) Recommendations for PHARM-DC improvement. The themes, as well as associated CFIR domains and constructs can be found in Table 2. In the following section, themes (with CFIR constructs italicized in parentheses), and representative quotes from pharmacists at Site A (SAP) and Site B (SBP) have been included.

**Table 2:** CFIR Domains and Constructs associated with Qualitative Themes and Subthemes

Theme	CFIR Domain	CFIR Constructs
1: PHARM-DC and Institutional Contexts	Intervention Characteristics External Setting Internal Setting Characteristics of Individuals	Relative Advantage, Complexity External Policies and Incentives Patient Needs and Resources Structural Characteristics, Culture, Compatibility, Available Resources, Networks and Communication Other Personal Attributes
2: Importance of PHARM-DC adaptability	Intervention Characteristics External Setting Internal Setting	Adaptability, Trialability, Complexity, Relative Advantage Patient Needs and Resources External Policies and Incentives Compatibility, Networks and Communication
3: Recommendations for PHARM-DC improvement and sustainability	Intervention Characteristics External Setting Internal Setting Characteristics of Individuals	Cost Patient Needs and Resources External Policies and Incentives Networks and Communication Structural Characteristics Available Resources Readiness for Implementation Leadership Engagement Access to Knowledge and Information Self-Efficacy

### Theme 1: PHARM-DC and institutional contexts

At Site A, the size of the existing TOC program and staffing were consistently noted as facilitators for PHARM-DC implementation (*Structural Characteristics).* Pharmacists at Site A had specific TOC training and had worked in a TOC setting for a number of years (*Other Personal Attributes).* For Site A, intervention pharmacists felt the intervention was a modest increase in TOC services compared to preexisting service offerings (*Relative Advantage)*. *The PHARM-DC patients, I feel like they get a little bit more service than we would typically provide for the other patients [SAP4].*

Site A has a robust TOC program outside of the PHARM-DC intervention, which facilitated the intervention (*Available Resources, Culture, Compatibility.)* Recently, the TOC program received additional FTEs to support pharmacy technician lines within the overarching TOC program. Some intervention pharmacists at Site A reported that the intervention was supported by additional staffing and personal resources such as pharmacy students, residents, and technicians, with technicians assisting with medication reconciliation and technical intervention components focusing on medication access and cost. Pharmacy technicians involved in the PHARM-DC intervention were designated TOC technicians, who assisted pharmacists with technical TOC tasks such as completing prior authorizations for medications and contacting pharmacies to adjudicate potential insurance difficulties. *Whatever the issue is, the technician will triage it. If it’s as simple as, they can’t get medication from the pharmacy, the technician can resolve that issue on their own. If there are more questions about side effects and the patient is wondering if they should change their dose, then our technician will forward this call to one of our pharmacy residents. And pharmacy resident will address this question. They will call back the patient and if something needs to be done, they will touch base with physician and they try to get new prescription or modify regimen. So, the clinical portion goes to resident, but if some [task is] more like a technical [one], the technicians can do, they resolve it themselves [SAP2].*

At Site A, pharmacists suggested that newly adopted California state policy (SB 1254) may have contributed to their ability to focus pharmacist staffing on TOC initiatives such as PHARM-DC (*External Policies and Incentives). So, we’ve been able to expand, in part because of the state laws, SB1254. But comparing how even after that, we’ve been able to expand additionally when other hospitals are maybe only hiring techs and not pharmacists, or they might get one pharmacist, and we have our huge, wonderful team and get to do all these fun things [SAP1].*

Site B had fewer members of the TOC team, with intervention pharmacists having formal training and expertise in areas outside of TOC (*Structural Characteristics, Other Personal Characteristics)*. *The other thing, too, is, that no one here really has a background in transitions of care or chronic disease state management [SBP1].*

Pharmacists at Site B reported that organizational transitions and limitations in staffing made providing all components of the intervention to a large number of patients especially challenging, noting that the level of complexity of the PHARM-DC intervention made it difficult to adopt (*Complexity)*. *So, [REDACTED] and I are the only two full-time pharmacists. Typically, this is a rare day where we’re both staffing. But typically, most of the time, it’s just one. Then, we have one* per diem *pharmacists. We are training another* per diem*. But that’s also difficult because we run into issues of… continuity of care. At a certain point, you’re here for 1 day, I don’t really know these patients, it’s difficult [SBP2]. It’s generally one pharmacist at a time that wears many hats. We recently increased our staffing such that [REDACTED] and I can both be together on Sundays, out of the week [SBP1]. What makes this program, I think, difficult to adopt, is just the levels of complexity of it [SBP1]. So, for example, the last week, I’ve been in the vault. So then, today I’m here, staffing intervention. I think sometimes it’s hard to switch gears [SBP2].*

Site B previously had pharmacy technicians involved in a medication reconciliation program, but were not currently involved in the pharmacist intervention, leaving Site B TOC pharmacists to perform all clinical and technical intervention tasks (*Compatibility)*. While Site B did not use pharmacy technicians in TOC interventions, pharmacy students were available to conduct BPMHs in both control and intervention patients. *When we had a medication reconciliation service, we used to employ certain pharmacy technicians on top of pharmacy interns, and they consistently provided good work. We had no objections to them [SBP1].*

Despite the size and training of the TOC team at Site A, intervention pharmacists still struggled to complete some intervention components. Pharmacists consistently reported difficulty contacting patients for the post-discharge follow-up call, which pharmacists attributed to inconsistencies in notifying the patient of the upcoming post-discharge call during hospitalization and identifying whom to contact. *The other pharmacists I talked to, they shared the experience that they have trouble reaching patients, so they call them, but they’re not able to reach anyone [SAP2].*

Additionally, Site A pharmacists suggested that many intervention components were more challenging due to the variety of patient needs and available resources. Site A pharmacists reported patient experiences that included language barriers and the need for translators to assist in delivering intervention components, including motivational interviewing (*Patient Needs and Resources)*. *I probably mentioned this already, but we do have a large portion of non-English-speaking population here in the hospital, too. So, there are times where we have to use, either in-person or over the phone interpreter in order to communicate to these patients [SAP5]. Multiple reasons, one, obviously for language barriers, when we have to use an interpreter. It’s hard to tell, but sometimes even if you’re trying motivational interviewing, I don’t know if the interpreter is interpreting it the exact same as the way I’m asking it, so I would say when patients are not English speaking [SAP7].*

Site A also has an automated post-discharge follow-up phone call system (CIPHER) which was not a component of the intervention, but all patients who are discharged from the hospital receive Site A pharmacists suggested these phone calls may be associated with their difficulty in reaching patients with post-discharge follow-up calls. *We do a post-discharge follow-up call on them and they’re like, “Three people have called me already,” probably the surgeon’s office, then the CIPHER call and then us calling them to see if they’re doing okay. So yeah, there’s definitely some overlap there. Everyone gets a CIPHER call [SAP3].*

Site B had fewer struggles contacting patients after discharge, as a checklist developed by a TOC pharmacist emphasized the importance of notifying the patient in the hospital that they would be receiving a post-discharge call. *We have a checklist of things that we discuss with the patient. One of the things that we discuss is, we let them know that we’d like to call them when they leave the hospital, we get their consent. If someone helps them with their medications, we might call them instead, whoever is the point person. We confirm with them the phone number and what time of day works best for them and let them know that a phone call’s coming. So, we find that cold calls, we don’t get as much response rate back [SBP1].*

Both sites emphasized the importance of networks and communication, specifically pharmacist relationships with prescribers to improve recommendation uptake (e.g., to modify medication regimens to reduce polypharmacy or otherwise improve the regimen’s appropriateness). Pharmacists reported that prescribers with familiarity with the TOC service and team, as well as a direct line of communication, improved the intervention pharmacist’s ability to complete intervention components (*Networks and Communication)*. *Definitely, there’s variation of how [providers] respond to our recommendations. I found doctors respond better if you already have a working relationship with them, if they know you, and they worked with you in the past, they are more open to accept your recommendations because trust is present already [SAP2].*

Pharmacists at Site A frequently reported that some prescriber groups were familiar with pharmacist TOC activities, but challenges remained with prescribers outside of the institution (e.g., private hospitalists or other physicians). *I would just say, which is going to be a barrier potentially anywhere is how certain providers are more open to interventions from pharmacy* versus *others. I think we have a pretty good team of hospitalists. If there’s a hospitalist involved, then they’re usually pretty good at making these changes that we recommend, but if there’s a private physician, some of them are a little bit more apprehensive [SAP4].*

Site A Pharmacists also reported that patients with a strong relationship with their PCP were more resistant to PHARM-DC intervention activities (i.e., to make changes to their preadmission medication regimen). *There are times where patients have relationships with our outpatient providers where…we only know them throughout their hospitalization, they’re a little bit more resistant I would say to interventions or changes that they’ve worked closely with their primary care doctors or outpatient providers with [SAP7].*

### Theme 2: importance of PHARM-DC adaptability

Intervention pharmacists at both institutions emphasized the importance of intervention adaptability. At Site A, a note template was adapted and used to document intervention delivery in addition to a Microsoft SharePoint site, which was created and adapted to streamline the intervention and track and communicate patient progression throughout their hospital stay (e.g., between different pharmacists caring for the same patient). *We had one template and then a couple months ago, we started to make recommendations to improving it to make it easier and to make documentation faster. And so, I think what we have now is a shortened version and it gives all the information we need to give. So, I think now the note is better [SAP3].*

Alternatively, pharmacists at Site B described a checklist used to guide intervention delivery and the use of “iVents” within the electronic health record to collect data and patient information needed to improve the yield of a variety of intervention components and communicate the tasks completed and still required by other intervention pharmacists (*Adaptability, Trialability)*. *One aspect of the pre-discharge interview that I forgot to mention, so when we touch down with the patient in a perfect world, if we’re able to see them in the hospital, at least, once while they’re in the hospital, and during the initial touchdown with the patient, we have a checklist of things that we discuss with the patient. One of the things that we discuss is, we let them know that we’d like to call them when they leave the hospital, we get their consent [SBP1].*

Pharmacists at both sites emphasized they are often had difficulty reaching the patient prior to discharge due to the timing of discharge and not wanting to delay the discharge process (*Compatibility)*. There were inconsistencies in pharmacist perceptions of when the best time was to provide education, suggesting that flexibility was required for when pharmacists were providing patient education (*Adaptability)*. Several pharmacists felt that the day of discharge was not the best time to provide medication education based on the specific needs and resources available for each patient (*Patient Needs and Resources)*. *I think for part of the study, we agreed that we’re going to do med review, med reconciliation at discharge and patient education, and then when we do phone call, we reinforce those education points that we provided. Sometimes it’s not always feasible, appropriate, or time permitted to do education at discharge, for different reasons. So, then I’ll focus more and spend more time on education to provide their family members after discharge, or even before discharge, but over the phone. Maybe not every patient requires education at the time of discharge. We might need to do that post discharge, and just at discharge make sure that actual, the discharge med list is correct [SAP2]. It’s a heated debate about patient education. So, is patient education at the time of discharge a good idea or not? Are patients receptive to the information or does it get glossed over in the 50 other pieces of paper the nurses give them? [SAP8].*

Further, there were additional variations between sites. Site A used an icon within the electronic health record to notify pharmacists when a patient was close to being discharged, prompting the pharmacist to complete the discharge medication reconciliation process and provide patient medication education prior to leaving the hospital. Site B pharmacists typically saw patients earlier in the hospitalization, focusing on medication regimen review and past problems with medication adherence, rarely seeing patients closer to discharge and deferring discharge medication reconciliation and patient education to the post-discharge call. Site B pharmacists suggested these differences could be due to the perceived value that seeing patients earlier in the hospitalization may have on TOC initiatives and the patient-pharmacist relationship. Additionally, Site B pharmacists reported that ordering clinicians often did not write discharge orders until immediately prior to patient discharge, leaving them with less time for medication reconciliation and discharge counseling prior to discharge (*Compatibility, Networks and Communication)*. *We don’t know if a discharge is happening until an hour before and then the whole team is reacting [BP3]. Going over their discharge summary, because we know that patients do not recall a lot of the information that’s explained to them at the point of discharge [BP1].*

The intervention at Site B was further adapted to accommodate a large number of patients who were discharged to skilled nursing facilities (*Adaptability)*. This involved contacting the skilled nursing facility and engaging facility staff with discharge processes and recommendations (i.e., identifying any discrepancies between the hospital discharge medication list and the medications administered at the facility). This adaptation was facilitated by existing networks, relationships, and communication between Site B and the skilled nursing facilities (*Networks and Communication)*. *So, some of our patients get discharged to skilled nursing facilities or rehab... In those cases, since someone’s ordering their medications and administering their medications, our process is a little bit different on the post-discharge side. We will call the facilities, ask to be redirected to the patient’s floor nurse, explain who we are and why we’re calling. Essentially, we first want to compare our discharge summary or our validated discharge medication list against the actual medication administration record at the facility just to make sure the discharge medication reconciliation was appropriate [SBP1].*

While study pharmacists at both sites noted that the intervention contained a large number of intervention components, Site B pharmacists specifically emphasized the complexity of the PHARM-DC intervention compared to other programs (*Complexity)*. Limited by staffing constraints, Site B pharmacists inquired if the intervention might benefit from additional adaptation to focus on further stratification of risk and associated intervention delivery in high-risk patients given the difficulty of offering all intervention components to a large patient population. *What makes this program, I think, difficult to adopt, is just the levels of complexity of it. I know [REDACTED], down the street, has a similar service to what we have. It’s called the red something. But they target a very small subset of very high-risk patients, and it’s very, less patients, but patients that require a lot of steps to help them get them on their way out. The only thing that I wonder is, if we’re not able to see that many patients, because we’re doing so many things per patient, is that still going to have the return on investment that perhaps a med rec service would have? [SBP1].*

### Theme 3: recommendations for PHARM-DC improvement and sustainability

Pharmacists at both sites had a wide variety of recommendations for intervention improvement, as well as statements on what would need to occur or be observed to assure intervention sustainability. Site A pharmacists provided a number of recommendations to improve care transitions related to medication discrepancies and errors, including developing protocols, collaborative practice agreements, and medication substitution policies that would allow for medications prescribed inpatient to be adjusted more efficiently (*External Policies and Incentives, Structural Characteristics)*. *One thing that they are trying to push through is reverse therapeutic auto[substitution] which will be very helpful for us. If something was therapeutically substituted inpatient and they erroneously continued that on discharge, we would be able to switch it back automatically” [SAP5]. I guess another thing is contacting the doctor takes a while, so if we can increase the way we ... maybe if pharmacists have more privileges, some type of collaboration agreement where things could…not everything we need to contact the doctor potentially. That might save some time and we will be able to reach more patients [SAP3].*

Both sites stressed that pharmacist familiarity, communication, and support from prescriber/hospitalist groups and continuity with patients would improve intervention sustainability and effectiveness (*Networks and Communication)*. Site A pharmacists reported interactions with private attending physicians, which made making recommendations and delivering the intervention more difficult. *I usually find residents are easy to reach, because they’ll have pagers, it’s part of their learning experience, right? They have to respond to phone calls. I think it’s most challenging for some private doctors who don’t like to be told what to do, or they might have something in mind that they haven’t documented, and sometimes what they did will make sense once they explain their rationale, but because we can’t know what they were thinking and we only base our recommendations on lab values/notes that we read, we might not get a full picture and we might recommend something that maybe not always accepted, for a good reason. And I found that private doctors write notes that are not as complete as hospitalists. So, I think it’s easier with hospitalist followed by residents and the least easy is with private physicians who see those patients on an outpatient basis. That’s my personal experience [SAP2].*

Site B pharmacists did not mention interactions with private attending physicians, as most inpatient physicians are hospitalists; few primary care physicians admit their own patients. Pharmacists further recommended that increasing the continuity of pharmacist care would potentially improve the PHARM-DC intervention (*Structural Characteristics)*. *What if we focus on just general medicine and we get to know everyone on these gen med teams very well, and they learn to like us and appreciate the work that we do and they can vouch for us, is that going to be sufficient to get this program off its feet. Having interdisciplinary support definitely did make [the medication reconciliation] program more adoptable because we’ve had the program for 5 years now [SBP1]. I think it would be great if the same pharmacist can follow that patient throughout their whole stay. So, the pharmacist did the history, did the discharge and the post-discharge call because they know the patient the best. I think that would be ideal because right now, there’s several different people involved in those different services. So that would be ideal [SAP4].*

When workload became difficult to manage, Site A pharmacists suggested that having open communication and additional team support was essential to intervention sustainability (*Networks and Communication)*. *One of the really great things about our TOC team is that we work really well together. And it is a team. So even though we’re covering different units, it’s always a communication of hey, it looks like discharge orders just got placed for your patient on a different floor. Do you have time, or do you want me to do it? So even if it’s not my patient, then we’re making sure it gets done [SAP1]. I think we communicate really well. [redacted] is always on it, do you need help with this? Or if we can help each other out, we just see if it’s busy to work on PHARM-DC discharge because as we said, we have our other high-risk discharge from the floor and the stroke patients, as well. And so, I think we try to help each other out and check in with each other, message each other and say, “Oh, are you working on this patient? Do you need help?” To make sure that we don’t miss anybody [SAP3].*

To further promote sustainability, intervention pharmacists at Site A asked for flexibility in the number of interventions, where they could use clinical judgement to provide the intervention components, they felt would produce the highest yield on a patient specific basis. *I think we need to still be given the flexibility to choose how that post discharge follow-up goes. Is it really necessary for every patient, or can we identify specific patients where we think they’re higher risk, and so we spend more time with them? Right now, if you compare it to our other post discharge calls, we call them, if they have a bad [health literacy] score, no matter what, even if they end up with no medications on their list when they’re discharged because of changes that happen, or maybe they don’t need things [SAP1].*

As noted above, Site A pharmacists reported that patients with existing relationships with primary care providers (PCPs) were more resistant to the pharmacy-provided interventions, making the intervention more challenging to implement and execute for these patients (*Patient Needs and Resources)*. Additional pharmacist conversations with PCPs and patient MI may be possible solutions to patients resistant to medication changes initiated by the PHARM-DC TOC pharmacists. *I think the most challenging thing for outpatient is when... I think it’s most challenging when patients don’t have enough trust in their hospitalist or hospital doctors, and they just want to follow the instructions from their PCP. I think that’s hard to overcome because it requires a lot of counseling, and we don’t have enough time to establish this trusting relationship [SAP2].*

Pharmacists at both sites had an appreciation for how MI positively impacted the intervention. Despite this appreciation, intervention pharmacists had difficulty consistently engaging patients with motivational interviewing, commenting they were uncertain the techniques they were using were correct, asking for additional training, and emphasizing that time constraints made the motivational interviewing process more difficult to sustain within the intervention (*Self-Efficacy)*. *…especially right after we finished the [motivational interview] trainings and feeling like it definitely made a difference in the tone of conversation with patients, so I would say that’s held true. It’s nice to feel more collaborative and not quite so prescriptive in our discharge education. That’s another thing that we’ve been trying to do, that we had talked about in the ideal state [SAP1]. I don’t think all patients need motivational interviewing, but the patients who do need it, I do try to provide it. It does take some time. I did motivational interviewing 2 weeks ago on a patient and it did take 20–30 min to complete it [SAP3]. There are variables that we can’t predict until we see the patient. For example, if they’re going to need motivational interviewing, if they’re going to need 5 min on the phone* versus *45 min on the phone, those things definitely limit our efficiency [SBP1].*

In some instances, pharmacists had patients that denied difficulties taking medications as prescribed which made identifying opportunities for MI more challenging. As such, pharmacists suggested that additional practice, training, and resources on MI may provide benefit for pharmacists providing the PHARM-DC intervention (*Available Resources, Access to Knowledge and Information). [The trainer] can effectively do motivational interviewing in like 2 min [and that it is] as effective as a pharmacist who’s on the phone for 17 min. And so, [the trainer] emphasized that it takes a lot of practice, and I think that that’s something that needs time to develop. I don’t think all patient encounters can be done in 2–5-10 min [SAP7].*

Pharmacists at Site B reported MI behaviors and techniques without specifically referring to these processes as motivational interviewing. *Especially, if the patient is not willing to stop their benzo[diazepine], there’s no moving it off of their medication list because it doesn’t change anything. What we will do is, we’ll talk to the patient in advance if we’re going to make a change like that and say, “Hey, you’ve been taking Lorazepam to help you sleep, but you still aren’t sleeping and you’re not feeling that great and it’s not doing anything, do you want to try melatonin and see how you feel on that while you’re in the hospital? Or, trazadone might give you less side effects, you can give it like a little try and see, if you wanted to try something else.” They’re usually amenable when you frame it like that,* versus*, we’re taking this off for your own good. So, we try to figure out what the issues are and see if they’re open to trying something new. So, it’s highest yield if we get them to try it in the hospital before we send them home, but otherwise we would defer it out to outpatient, for a lot of things [SBP2].*

Both Site A and Site B pharmacists agreed that leadership engagement and institutional support are required for intervention sustainability (*Leadership Engagement, Readiness for Implementation)*. Pharmacists at both sites proposed that both rich, detailed stories of how the intervention prevented dangerous medication misadventures and presentation of quantifiable outcomes were important. Pharmacists and pharmacy leadership frequently stressed the importance of sharing important pharmacist interventions within committees and meetings that included institutional leadership and members of the interprofessional care team. Further, pharmacy leadership at both institutions noted the importance of emphasizing the potential severity of medication-related errors and how the intervention prevented the errors from occurring. *We have been able to show the percentage of patients who have at least one serious or life-threatening intervention drug-related problem at discharge that we identified and resolved. Then those are the stories and those are what sticks with everybody [SAP8].*

Tangible outcomes that pharmacists felt were important to justify longitudinal support for the PHARM-DC intervention included reductions in readmissions, improvements in patient satisfaction scores, quantification of preventable medication errors, and return-on-investment calculations to justify additional staffing (*Cost)*. With specific and tangible outcomes highlighted and presented, Site A appeared relatively more successful at leveraging data and anecdotes for additional staffing and pharmacy FTEs. *I drive my people crazy with data… The transitions of care – before even I was in this role – this was something that was felt to be a goal of the medical center. My initial FTEs that I got back then was because we did a study that was a quality mission priority to the organization that demonstrated that even with a pilot study, we made a significant impact on. There was an impact on readmissions.... We worked with the physician and health services researcher, and we published it. It was published in one of the Accountable Care journals several years ago. That study data did go all the way up the C-suite…. I got three pharmacists from that because of that study. The C-suite was interested now because readmissions were a priority [SAP9]. Well, I think our world is still financially driven. If we can show that there is benefit in terms of readmission rates, patient satisfaction scores. I think that we have to think about how we can utilize our resources to maximize returns on the money we spent on pharmacists and technicians. And that will be, I think, more accepted by management. Because our programs are expensive. So, we have to show how can we make more of a difference for less cost [SAP2]. I’m not in a position to win this thing. That’s the problem. So, everything comes down to beauty is in the eyes of the beholder. Make up a million end points and report all the positive ones. Whether it’s readmission, patient satisfaction, tangible, intangible. I come back to the same discussion, I sit in on the budget meetings, and it’s return on investment. If I’m paying out $150 thousand in salary, what am I getting back? There isn’t a single service line here or physician that would turn down a pharmacist [SBP4]. So, I think, for our hospital, we have a passion for lots of clinical pharmacy pilots that never seem to take off. I think it’s just, we’re unable to show the hospital the return on investment. Our medication reconciliation program was originally based in the emergency department, and our data was very good. However, we didn’t have enough interdisciplinary support just because we were doing a couple patients from oncology, a couple of patients from ortho. So, no one really saw the impact of what we were doing. So, we then said, okay, well, even though we’ve shown the return on investment, and we’ve shown error reduction, the hospitals still not taking us up [SBP1].*

## Discussion

The PHARM-DC intervention was successfully initiated at both institutions involved in this study. Prior emphasis on TOC initiatives, TOC staffing, and pharmacist background in TOC at Site A improved implementation and likely sustainability of the PHARM-DC intervention. Site B had fewer intervention pharmacists and resources committed to the PHARM-DC intervention, with intervention pharmacists having less TOC training and experience than pharmacists at Site A. Both sites found opportunities to improve the intervention by adapting pharmacist documentation materials and developing an intervention checklist to assure that intervention activities were documented appropriately, and that patients were receiving all of the intervention components. Pharmacists at both sites provided a number of suggestions for intervention improvement and suggested that intervention sustainability depended on tangible outcomes that resulted in improved patient care and had positive financial returns for their respective institutions in order to gain the support of institutional leadership.

When considering the variations in implementation across sites, there are a number of key elements which require further consideration. First, the site-specific and institutional contexts appeared to vary based on the effects of external factors (Outer Setting) on internal environment (Internal Setting). In addition to an existing emphasis on TOC programs, major policy reform introduced and passed in California (SB 1254) may have positively affected BPMH and TOC workflow and staffing at Site A. This legislation “requires a pharmacist at a hospital pharmacy to obtain an accurate medication profile or list for each high-risk patient upon admission of the patient under specified circumstances, authorizes an intern pharmacist or a pharmacy technician to perform the task of obtaining an accurate medication profile or list for a high-risk patient if certain conditions are satisfied, and require the hospital to establish criteria regarding who is a high-risk patient for purposes of the bill’s provisions and determine a timeframe for completion of the medication profile or list, based on the populations serviced by the hospital [24]. With increased FTEs dedicated to TOC programs, a greater number of technical TOC tasks were delegated to pharmacy technicians specifically dedicated to the TOC program, such as BPMH on admission and completion of medication prior authorizations, allowing TOC pharmacists to focus on patient and caregiver counseling and education, medication regimen reviews, and recommendations to physicians. At Site B, there were no technicians dedicated to the technical tasks of TOC and BPMH and fewer pharmacists working specifically and exclusively on TOC initiatives (although pharmacy students were available to take BPMHs at both study sites). As a result, intervention pharmacists at Site B spent considerably more time involved in technical components of the PHARM-DC intervention and were required to complete all technical and clinical elements of the PHARM-DC intervention. In the existing literature pharmacy technicians may play an important role in TOC programs, with TOC programs employing pharmacy technicians in traditional and novel roles to expand TOC programs [25–27]. Most consistently, pharmacy technicians have been used to collect medication histories and reconciliation, with technicians completing these activities successfully in a number of interventions [28–30]. While the PHARM-DC intervention does not explicitly require or recommend pharmacy technicians or additional support personnel to complete intervention components, the inclusion and training of such individuals may provide a cost-effective alternative to some pharmacist-delivered intervention components within the PHARM-DC intervention [30, 31].

Further, specific training in TOC appeared to be present for most intervention pharmacists at Site A, which may facilitate intervention adoption and sustainability. Specific TOC training was not present in Site B, and while limited by staffing constraints, Site B intervention pharmacists may have felt the intervention to be more complex or difficult to implement due to less familiarity and training with TOC. In addition to specific training and lack of TOC background, limited staffing and competing responsibilities are likely to contribute to Site B pharmacists’ perception of intervention complexity and difficulty increasing the volume of patients they were able to provide the PHARM-DC intervention for.

Further, while staffing concerns and competing responsibilities are likely to require organizational change, additional TOC training may help to improve pharmacist-led TOC interventions when pharmacists providing the interventions have not obtained specific training prior to program initiation [32, 33]. At Site A, most pharmacists received this training during postgraduate education, but training could be provided during pharmacy school or in professional development programs. While no current standards for TOC education currently exist, pharmacy schools and residency programs have increased their focus on TOC training in recent years [34–37]. Pharmacists providing TOC services may benefit from formal training and/or background in TOC to improve intervention delivery and sustainability.

Pharmacists’ relationships and familiarity with hospital physicians and physician groups proved to be critical for intervention success. When pharmacists had familiarity and open lines of communication with physicians, medication changes and recommendations were more readily accepted. Timely communication appeared to improve the efficiency of pharmacists providing the intervention, potentially increasing the number of accepted recommendations and the volume of patients each pharmacist was able to see. Intervention delivery may be less challenging at institutions with a larger proportion of dedicated inpatient physicians (hospitalists and residents) due to their familiarity with pharmacy interventions and accessibility. Conversely, the intervention may prove to be more challenging when hospital systems have a large number of private physicians who spend little time in the hospital. Timely and effective communication between providers is an important element of TOC interventions, with inadequacies in communication noted as potential barriers to successful TOC intervention implementation and sustainability [38, 39].

In addition to continuity and familiarity between pharmacists and physicians, there may be benefit in patient-pharmacist continuity. Pharmacists at Site A reported that while having more pharmacists dedicated to TOC initiatives may increase the number of patients who receive these services, a team-based approach may potentially decrease the continuity of care that patients otherwise receive from a single intervention pharmacist. Pharmacists who participated in interviews at Site A emphasized that while staffing concerns were less prevalent, continuity and having a single pharmacist follow patients throughout their stay may improve the intervention.

One of the most noticeable barriers for successful intervention implementation at Site A included language barriers between pharmacists and patients/caregivers. Site A is located in a state where more than 20% of the population is considered to have Limited English Proficiency (LEP), suggesting that while it may be potentially more difficult to implement a TOC program, it is still attainable. Implementation of the PHARM-DC intervention may be more resource-intensive in health systems with a high proportion of LEP older adults due to the additional time and resources required to provide discharge medication education and follow-up through an interpreter. Language barriers may be an important barrier to scalability and dissemination in other health systems with large proportions of patients with LEP. Challenges expressed by pharmacists around the difficulty of providing discharge education may be exacerbated by language barriers. While individuals with low health literacy are likely to benefit from patient-appropriate medication information delivered by pharmacists [40], older adults and their caregivers with LEP may experience specific benefits from additional medication management communication given lower health literacy and numeracy levels in this population if the information can be delivered in an appropriate way [41–43]. Discharge forms, medication package inserts, and pharmacy instructions often include language at a high reading level, increasing the risk of medication-related errors and ADEs among older adults with LEP [44]. A number of recommendations exist to promote effective transitions of care for patients with LEP including but not limited to; providing trained interpreters rather than relying on rudimentary language skills or patient’s family member and providing written instructions in the language preferred by the patient [45, 46]. The PHARM-DC intervention may benefit from the design of additional procedures and processes to accommodate the language barriers that patients and providers may experience within the TOC process. This may be especially true for implementation sites which serve a large proportion of patients with LEP.

### Limitations

This study has a number of limitations which should be considered. First, there were fewer pharmacists at Site B involved in and providing the PHARM-DC intervention, limiting the number of interviews that could be completed. However, all pharmacists involved in the intervention at both sites contributed to the study. Additionally, interviews were completed over the course of 6 months while the intervention was being delivered. As a result, interviews were completed at varying stages of intervention implementation which may have influenced the responses provided by pharmacists during interviews. Further, COVID-19 impacted the delivery of TOC interventions throughout the duration of the study period, which may have influenced intervention pharmacist perspectives of the intervention in the context of changing protocols and workloads. Lastly, interviews were completed with pharmacists at two institutions, limiting the generalizability of the results.

## Conclusions

The PHARM-DC intervention can be successfully implemented in medical institutions with considerable variation in staffing, training, and patient populations served. Despite successful implementation, intervention pharmacists believe there are a number of opportunities to improve the intervention focusing on efficacy, scalability, and sustainability. Future work should focus on the clinical outcomes and cost-effectiveness of the PHARM-DC intervention to improve sustainability and uptake.

## Supplementary Information

**Additional file 1.** Pharmacist Interview and Focus Group Guide.

**Additional file 2.** Pharmacy Management Interview Guide.

## Data Availability

The datasets used and/or analyzed during the current study are available from the corresponding author on reasonable request.

## Abbreviations

ADE: Adverse drug event
PRCT: Pragmatic randomized clinical trial
PHARM-DC: Pharmacist Discharge Care
TOC: Transitions of care
CFIR: Consolidated framework for implementation research
MedBridge: Medication Reviews Bridging Healthcare
CMR: Comprehensive medication review
OPERAM: Optimizing Therapy to Prevent Avoidable Hospital Admissions in the Multimorbid Older People
OPTIMIST: Odense Pharmacist Trial Investigating Medication Interventions at Sector Transfer
MI: Motivational interviewing
BPMH: Best possible medication history
IRB: Institutional Review Board
PCP: Primary care provider
LEP: Limited English Proficiency
