# Supplementary material for: A qualitative dual-site analysis of the pharmacist discharge care (PHARM-DC) intervention using the CFIR framework
Source: BMC Health Serv Res. 2022 Feb 12;22:186. doi: 10.1186/s12913-022-07583-5 (PMC8840769; doi:10.1186/s12913-022-07583-5)
Supplement: Supplementary file 1 — Additional file 1. Pharmacist Interview and Focus Group Guide. [file 12913_2022_7583_MOESM1_ESM.docx]

**Additional file 1.** Pharmacist Interview and Focus Group Guide

**Moderator:** Hi. I’d like to thank you for your willingness to participate in our discussion. My name is [Facilitator] and with me today is [Note taker]. We work here at [Medical Center] in the [Department] Department.

I am here today to conduct an [interview or focus group] about your thoughts and perceptions surrounding the pharmacist-led hospital discharge intervention (Pharmacist Discharge Care (PHARM-DC) Study). We are also interested in your ideas about the pros and cons of the intervention, and ways to improve this intervention for you, your patients, and fellow providers.

The information you share with us will help us understand the barriers and facilitators associated with implementing this intervention and how to improve it.

**Expectations**

I’d like to tell you what to expect from today’s [interview or focus group].

We’ll be here for about an hour. During that time, I will ask you a few questions. There are no right or wrong answers to these questions. You are the expert, and we’re here to learn from *you*.

1. Your comments and suggestions will be used for improving this intervention.

2. You can pass on any question that you prefer not to answer, and you don’t have to answer any questions at all if you choose. In addition, if you do not have anything to share about a particular question then you may pass.

3. Please describe your experiences as candidly as possible. Your comments will only be used to improve the quality of this intervention at your site. We won’t personally identify you with anything you say.

4. We have plenty of time, but since we have a lot of topics, we may have to leave one topic to go forward to the next.

5. We will take some notes and use a tape recorder just to make sure we get all your comments, but the tapes will be erased after that.

6. You can ask for clarification at any time.

Before we get started do you have any questions for me?

**Ice Breaker**: Can you tell us about your day-to-day responsibilities and role at [Redacted]

What is your involvement in this intervention?

Let’s start with the discussion now.

1. Describe the intervention from your perspective. [Intervention Characteristics]
   1. What is the process for completing the intervention?
   2. What are the components of the intervention?
      1. Does everyone get cipher?

follow up with appts?

post rehab hard to follow up

who made these reports

1. Can you give some examples of situations where the intervention was difficult to complete? How about easy to complete?  Walk me through how the intervention worked for patients during these situations.  [Barriers and Facilitators]
   1. What made providing the intervention challenging?
   2. How could the process be improved?
   3. Documentation: how useful have the templates been? What would be ideal for documentation?

** describe your pharmacy dept set up

1. How has your regular workflow changed as a result of implementing the intervention? [Inner Setting]
   1. How did you manage workflow on busy days?
   2. How has workflow changed as a result of the intervention?

1. What components of the intervention do you think are most important? [Individuals Involved, Inner Setting, Process]
   1. How does your approach differ from other pharmacists providing the intervention?
   2. What about the place where you work has made it easy or hard for you to carry out the intervention?

1. What are the roles of the nurses and prescribers involved in the intervention? [Process]
   1. What has been your experience working with these two clinician groups? What has worked well? What has been challenging?
   2. What differences exist between outpatient and inpatient prescribers?
   3. Are there any differences between inpatient and outpatient pharmacists with what they focus on during the patient’s hospital stay?

1. Have you made a recommendation to deprescribe a medication or other recommendation to change the medication regimen? How did that go? [Benefits]
   1. How often are the recommendations being followed or accepted? How could this be improved? [Barriers]
   2. Are there differences between how the recommendations are being accepted between different provider types or groups? (e.g., NP vs Resident)

1. In an ideal world, how would the intervention work? [Process, Intervention]

1. Has time pressure affected your ability to perform this intervention? [Process, Intervention]
   1. Do you feel burned out? Due to COVID or for other reasons? How has the intervention been adapted for COVID? [adaptation]
   2. How do you balance the quality/thoroughness of the intervention with the number of patients you can see?
   3. Is this intervention sustainable? Why/ why not?
   4. What needs to happen for this to be sustainable?
      1. Pharmacy techs?
      2. Collect information to share with management? What data?

1. What else should we know about how you implement the Pharmacist Discharge Care (PHARM-DC) intervention at your institution
