# Supplementary material for: A qualitative dual-site analysis of the pharmacist discharge care (PHARM-DC) intervention using the CFIR framework
Source: BMC Health Serv Res. 2022 Feb 12;22:186. doi: 10.1186/s12913-022-07583-5 (PMC8840769; doi:10.1186/s12913-022-07583-5)
Supplement: Supplementary file 2 — Additional file 2. Pharmacy Management Interview Guide. [file 12913_2022_7583_MOESM2_ESM.docx]

**Additional file 2.** Pharmacy Management Interview Guide

- Ice Breaker: Please tell us about your day-to-day responsibilities and role at Cedars?

Now we want to understand a little more about the pharmacy setup at your institution.

- Describe how your pharmacy department is structured.
  - How many pharmacists do you have?
  - Where are the pharmacists positioned in the institution?
  - What is the breakdown of clinical, inpatient, and other care responsibilities?
  - Are there any ways in which your program is different from others?

- What is your familiarity with the Pharm DC intervention? What is your involvement?
  - Would you describe it for us?
  - Has it been adapted over time? How?
  - What are the most important parts?
  - What are the barriers to implementing the PHARM-DC intervention (resources, structural, etc.)?
  - What parts of the intervention are working well?  What about not so well?
  - How well does this intervention work compared to what other institutions are doing.
  - What would it take for the intervention to be sustainable? Both now and in the future?

- What are your current department’s ongoing priorities?
  - What are your current readmission goals or priorities?
  - How about current organization priorities or initiatives?
  - What current projects is your department currently working on? Resident, Organization, or otherwise?
  - What other programs overlap with the Pharm-DC intervention?
  - What data is needed to show admin the worth of transitional care programs?
- What is your perception of physician buy-in for the Pharm-DC program?

We understand there was a change in the policy SB1254 that pts; how instrumental was this policy

did the TOC change because of this bill? Admin changes, incentives?

How does this align with current priorities and future vision for pharmacist services?

Is there overlap with this intervention with current and future services?

Has anything been changed with the pharmacist role because of this trial?

What would it take to sustain this intervention/service

How much time can we afford per patient?

Is it feasible to do this for more pts?

Bill separately for this work?

What competing factors are there for maintaining this service

More patients and fewer components?

Is there overlap with this intervention with current and future services?

Has anything been changed with the pharmacist role because of this trial?

- What are the ideal transitions of care program?
  - What types of transitions of care programs do you wish you had here?
  - What are the barriers to getting the ideal transitions of care programs at your institution?
  - In your experience with transitional care interventions, what separates the ones that end up getting continued from those that fizzle out?

Anything else you would like to share about the PHARM-DC intervention or anything else that you thought of while we were talking?
